# Supplementary material for: Protein Modelling Highlighted Key Catalytic Sites Involved in Position-Specific Glycosylation of Isoflavonoids
Source: Int J Mol Sci. 2023 Aug 2;24(15):12356. doi: 10.3390/ijms241512356 (PMC10418691; doi:10.3390/ijms241512356)
Supplement: Supplementary file 1 [file ijms-24-12356-s001.zip › ijms-2499294-supplementary.pdf]

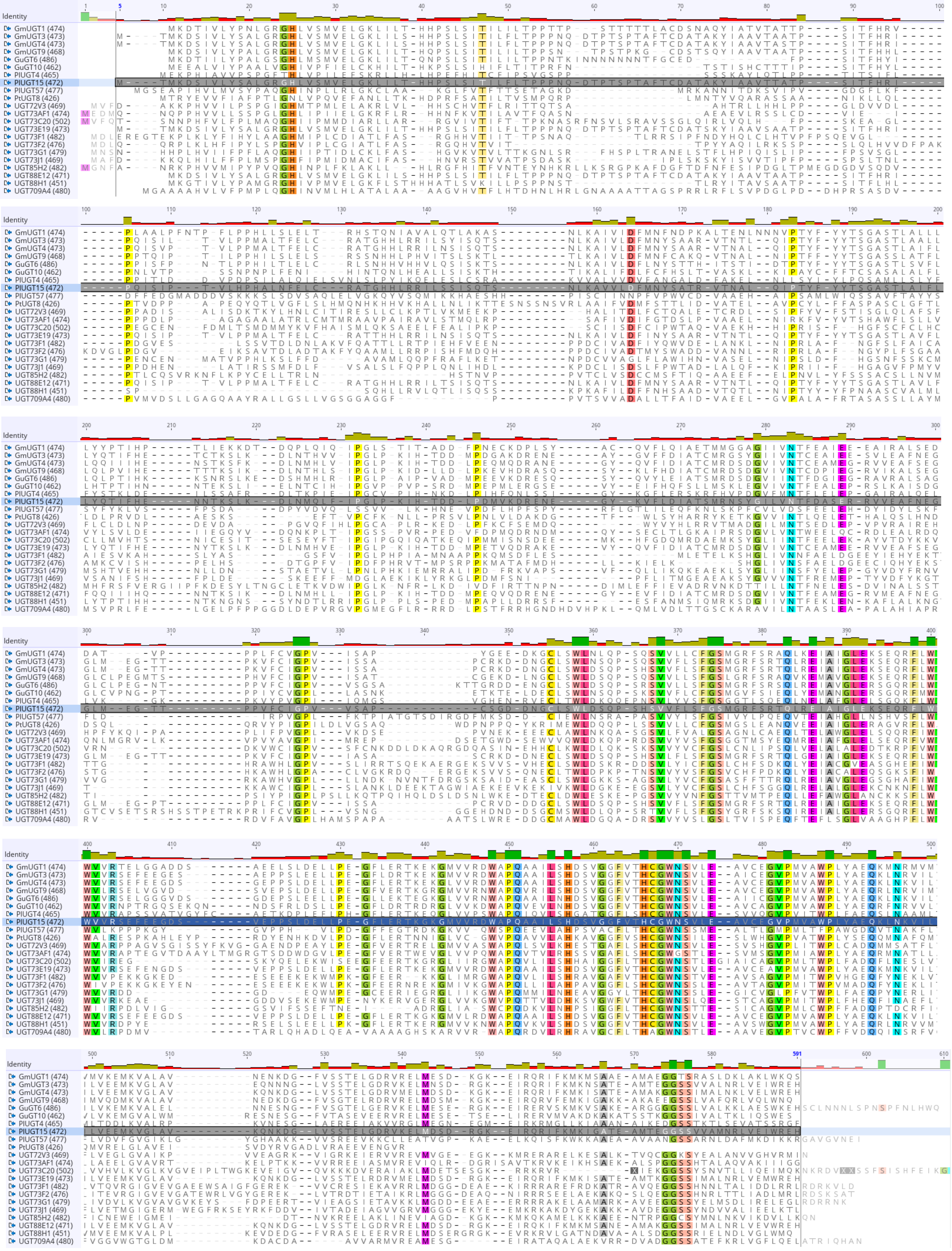

Figure S1: Multiple sequence alignment of selected UGTs. Amino acids with consensus (>85%) are shown in colors. PlUGT15, used as the model UGT, has been highlighted.

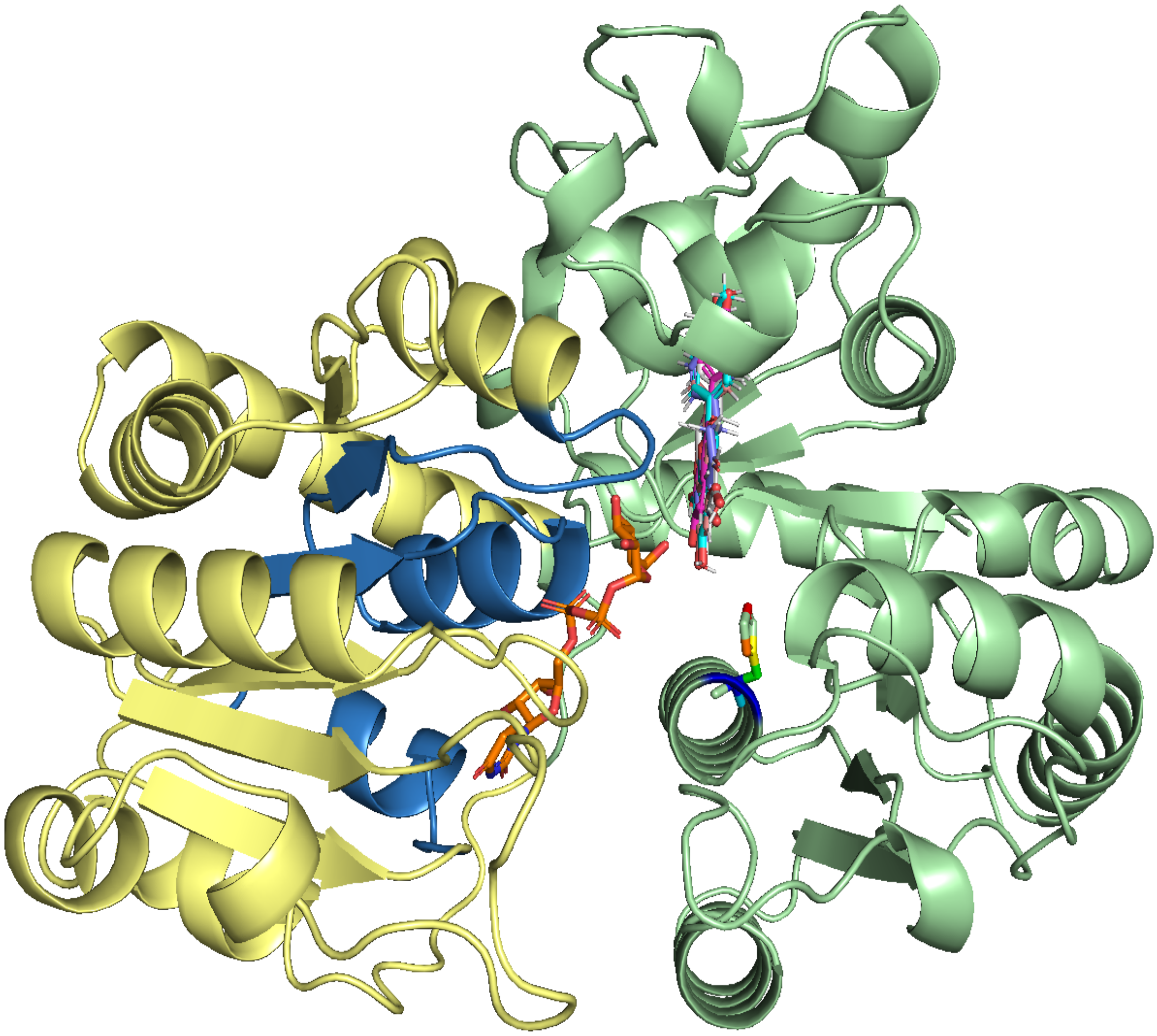

Figure S2: Protein model of PLUGT15 (developed with Alphafold2). The model has been shown as a carton. The N-terminal is coloured pale yellow, the C-terminal is pale green and PSPG motif is coloured marine. UDP-glucose, substrates and catalytic His have been shown in the stick model. UDP-glucose is coloured in salmon and O atoms are shown in red colour. Catalytic His is coloured in rainbow colours. The carbon skeleton of biochanin-A, daidzein, formononetin; genistein, liquiritigenin and naringenin is coloured cyan, pink, yellow, salmon, grey, and slate respectively, and the oxygen atoms are coloured red.

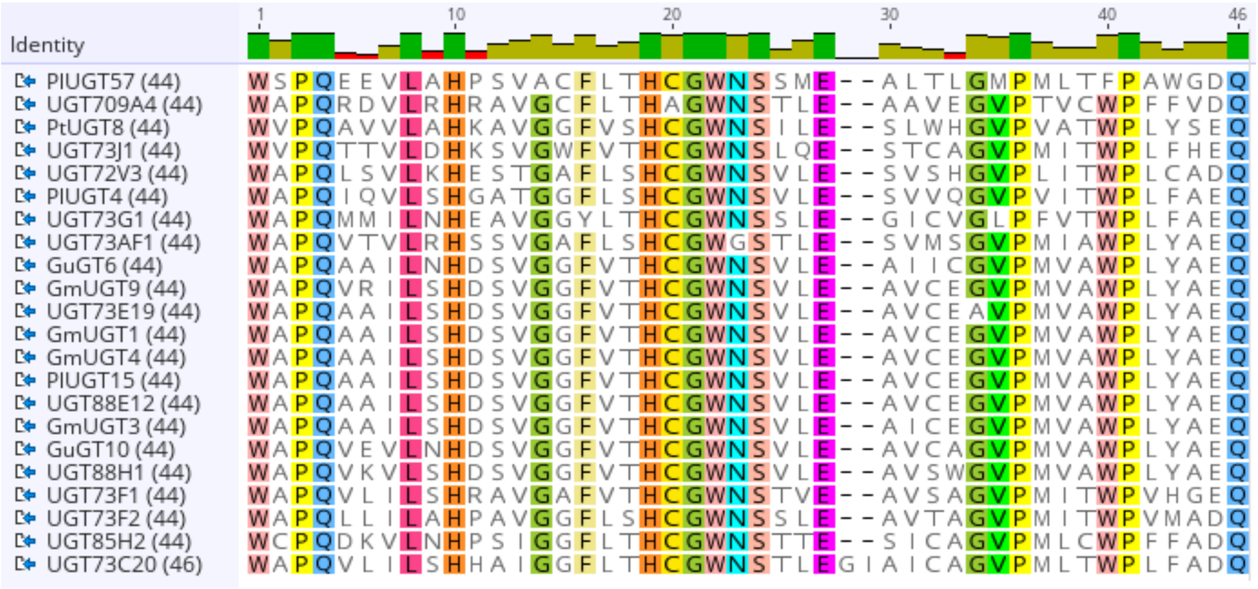

Figure S3: Multiple sequence alignment of PSPG motif of selected UGTs. Amino acids with consensus (>85%) are shown in colors.

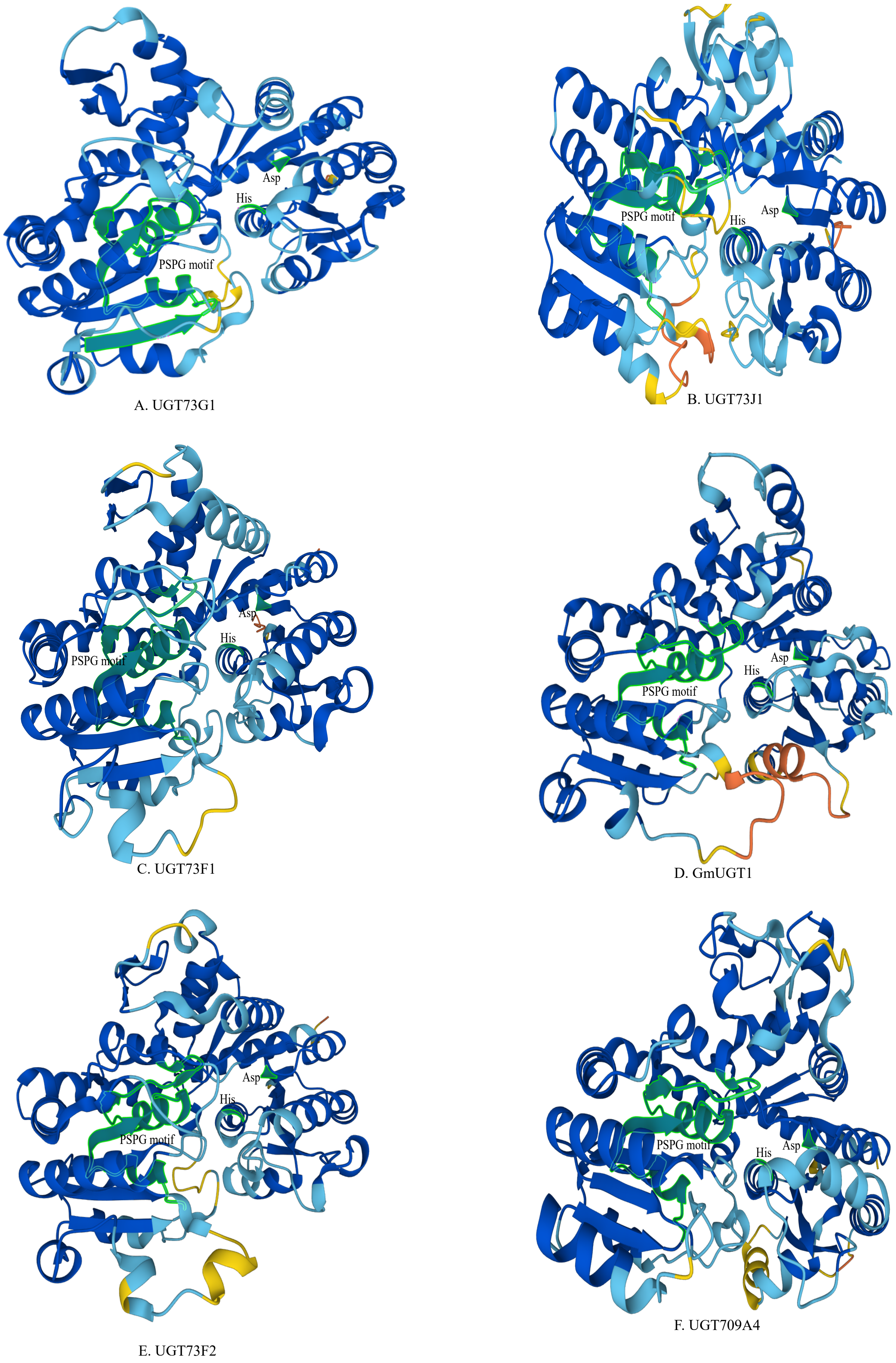

Model confidence	pLDDT score	A. UGT73G1			B. UGT73J1			C. UGT73F1			D. GmUGT1			E. UGT73F2			F. UGT709A4		
		His	Asp	PSPG motif	His	Asp	PSPG motif	His	Asp	PSPG motif	His	Asp	PSPG motif	His	Asp	PSPG motif	His	Asp	PSPG motif
Very high	90	Yes	Yes	Yes	Yes	Yes	Yes	Yes	Yes	Yes	Yes	Yes	Yes	Yes	Yes	Yes	Yes	Yes	Yes
Confident	90 > & <70																		
Low	70 > & <50																		
Very low	<50																		

Figure S4 (Cont.)

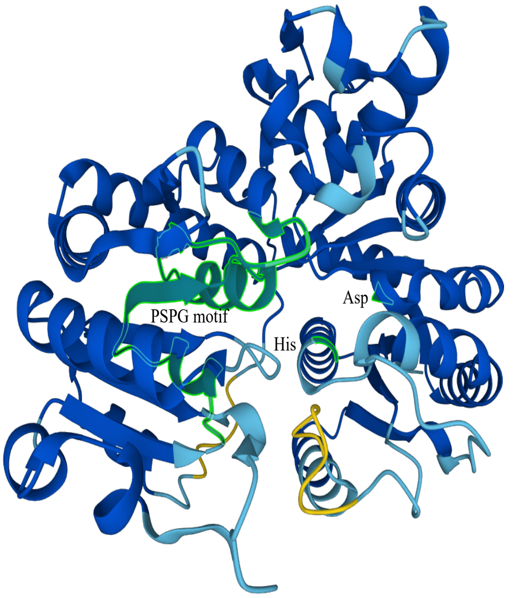

G. UGT88E12

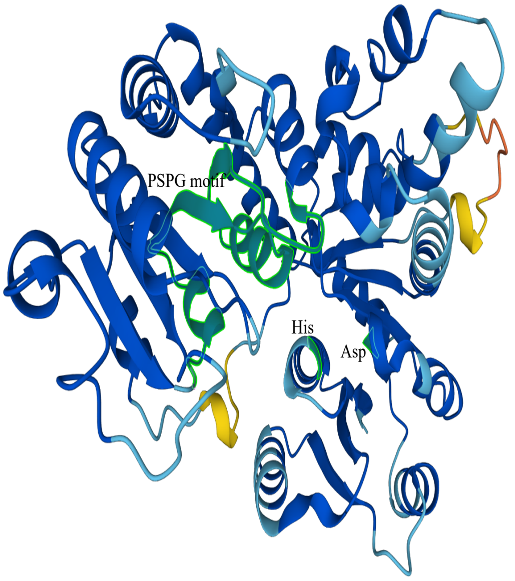

H. UGT88H1

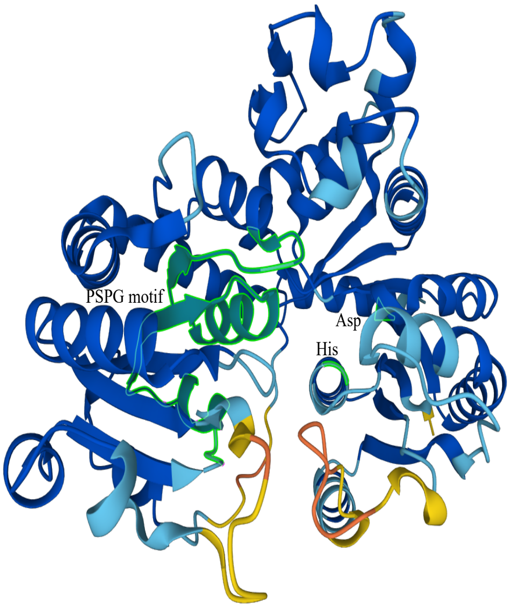

I. GmUGT3

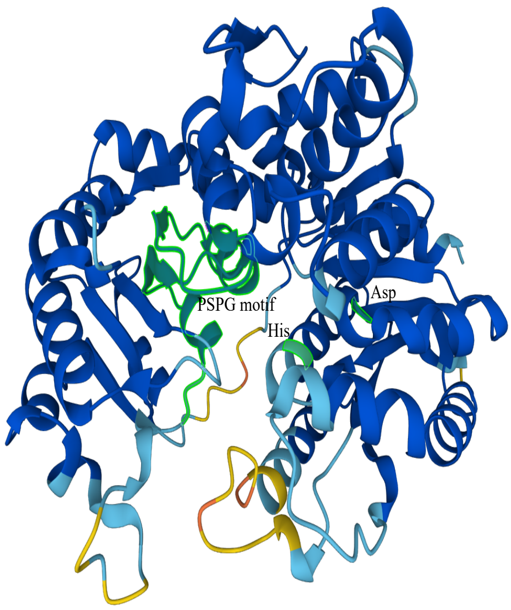

J. GmUGT4

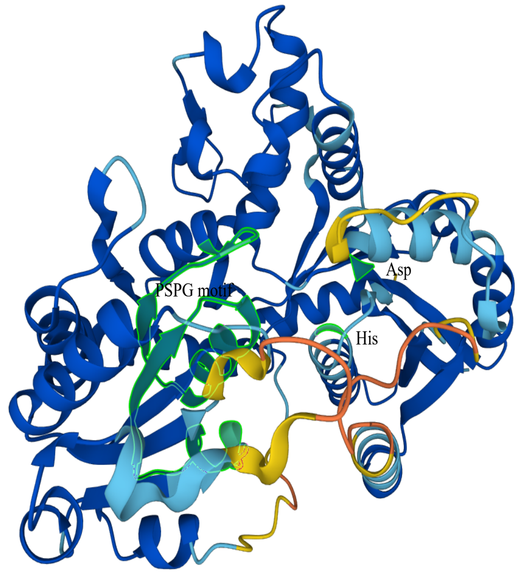

K. GmUGT9

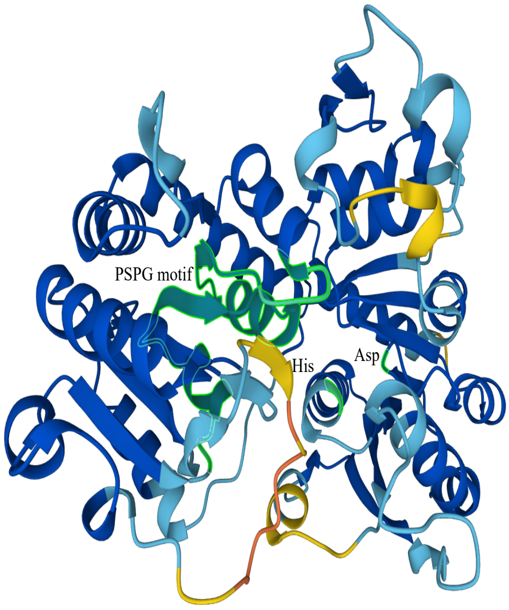

L. UGT72AF1

Model confidence	pLDDT score	G. UGT88E12			H. UGT88H1			I. GmUGT3			J. GmUGT4			K. GmUGT9			L. UGT72AF1		
		His	Asp	PSPG motif	His	Asp	PSPG motif	His	Asp	PSPG motif	His	Asp	PSPG motif	His	Asp	PSPG motif	His	Asp	PSPG motif
Very high	90	Yes	Yes	Yes	Yes	Yes	Yes	Yes	Yes	Yes		Yes	Yes		Yes	Yes		Yes	Yes
Confident	90 > & <70										Yes			Yes			Yes		
Low	70 > & <50																		
Very low	<50																		

Figure S4 (Cont.)

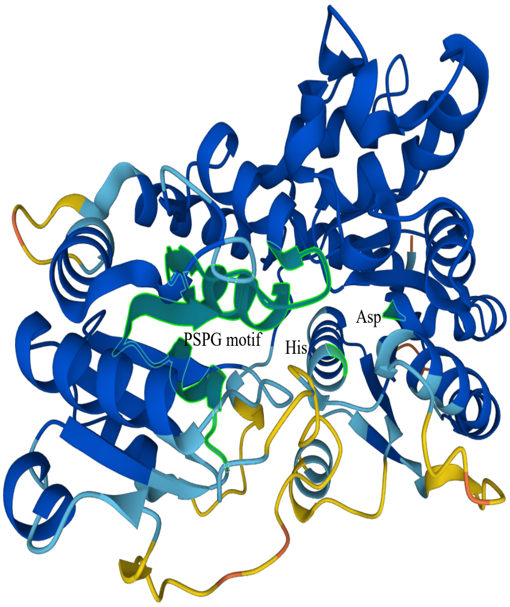

M. UGT72V3

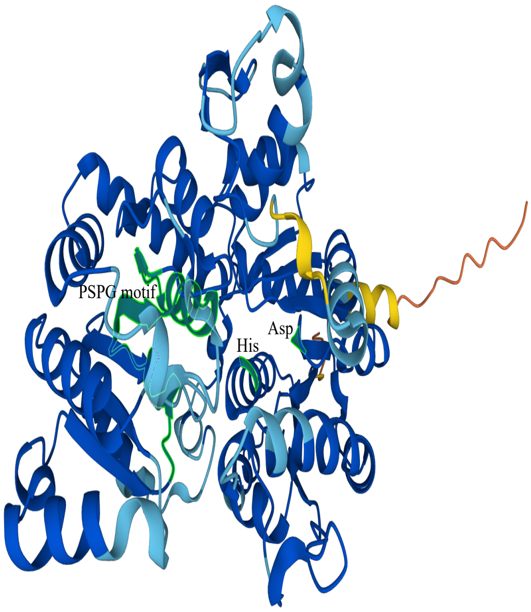

N. UGT73C20

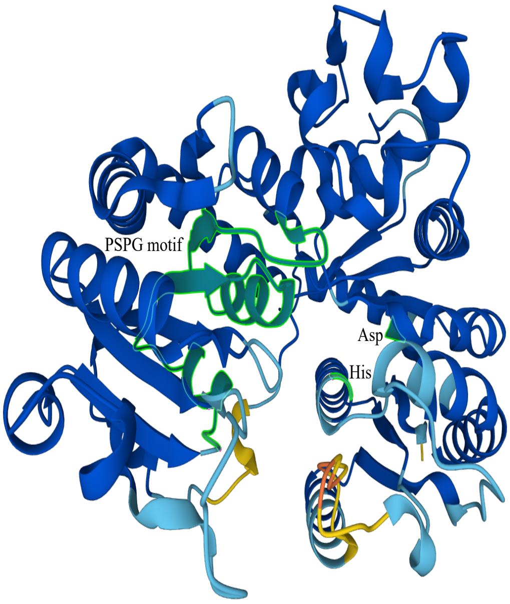

O. UGT73E19

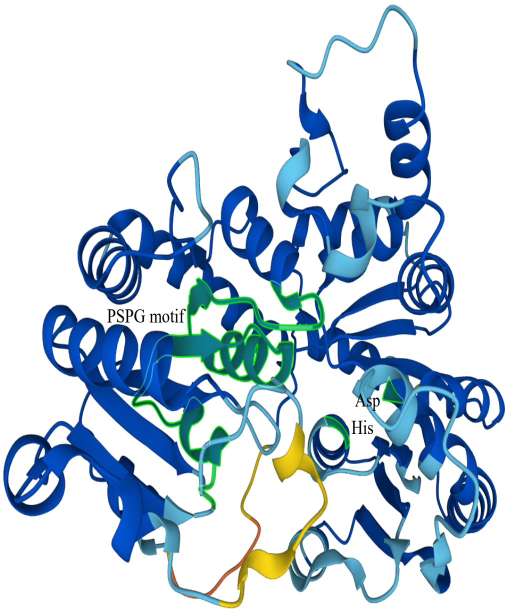

P. PlUGT4

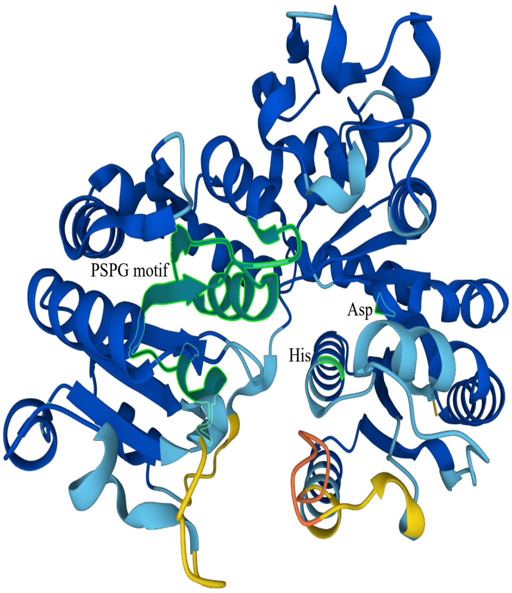

Q. PlUGT15

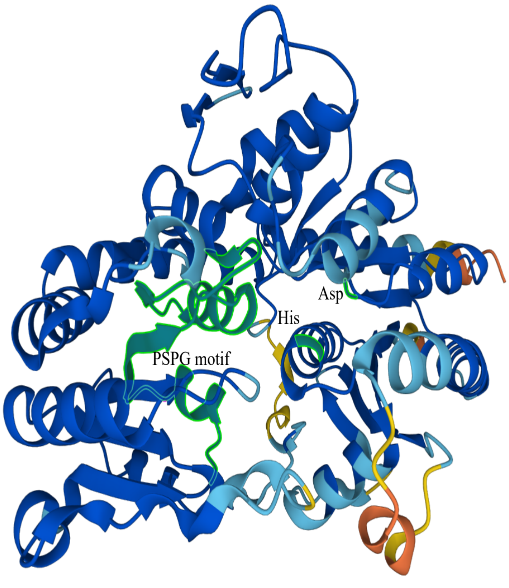

R. PlUGT57

Model confidence	pLDDT score	M. UGT72V3			N. UGT73C20			O. UGT73E19			P. PlUGT4			Q. PlUGT15			R. PlUGT57		
		His	Asp	PSPG motif	His	Asp	PSPG motif	His	Asp	PSPG motif	His	Asp	PSPG motif	His	Asp	PSPG motif	His	Asp	PSPG motif
Very high	90		Yes	Yes	Yes	Yes	Yes		Yes	Yes	Yes	Yes	Yes		Yes	Yes	Yes	Yes	Yes
Confident	90 > & <70	Yes						Yes						Yes					
Low	70 > & <50																		
Very low	<50																		

Figure S4 (Cont.)

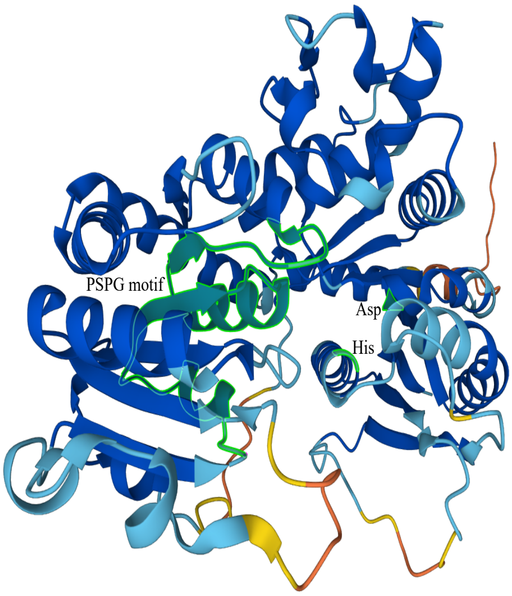

S. GuGT6

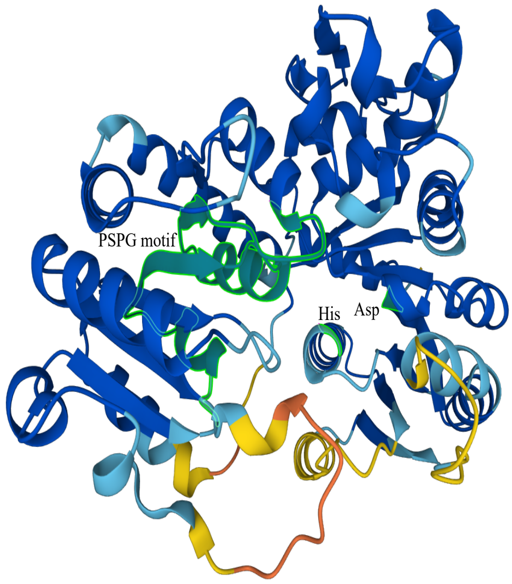

T. GuGT10

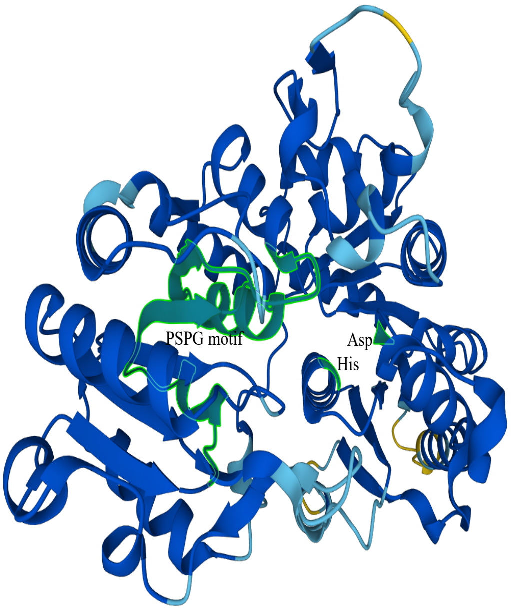

U. PtUGT8

	Model confidence	pLDDT score	S. GuGT6			T. GuGT10			U. PtUGT8		
			His	Asp	PSPG motif	His	Asp	PSPG motif	His	Asp	PSPG motif
	Very high	90	Yes	Yes	Yes		Yes	Yes	Yes	Yes	Yes
	Confident	90 > & <70				Yes					
	Low	70 > & <50									
	Very low	<50									

Figure S4. Protein models of selected UGTs developed with Alphafold. The protein models are shown as cartons and coloured as per pLDDT score: blue: >90 score (model confidence very high), cyan: >90 and <70 (model confidence confident), yellow: 70> and <50 (model confidence low), and orange: <50 score (model confidence very low). Catalytic amino acids (histidine and aspartic acid) and PSPG motif region are highlighted with green shade and their pLDDT score has been shown.

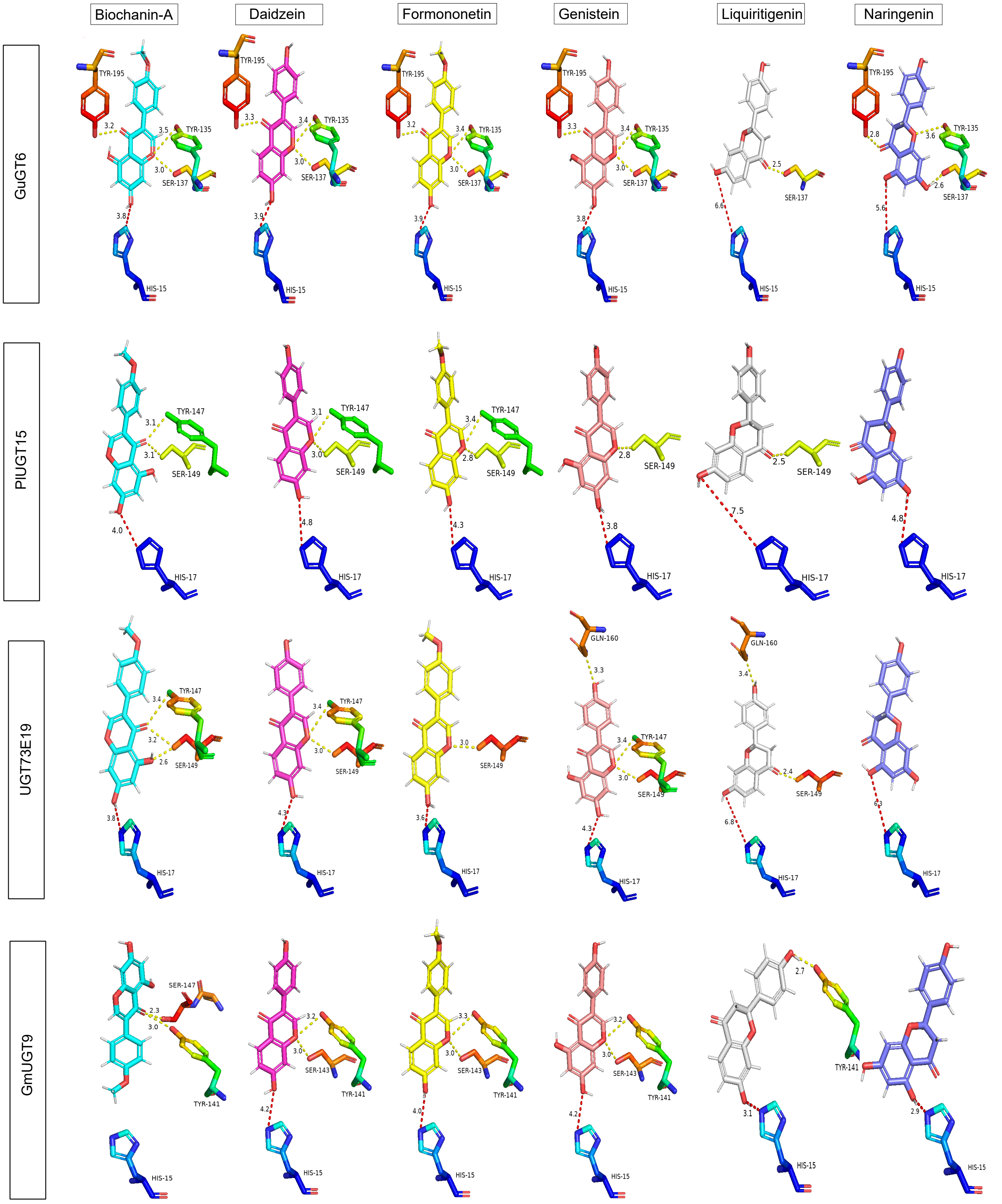

Biochanin-A
Daidzein
Formononetin
Genistein
Liquiritigenin
Naringenin
GuGT6
PlUGT15
UGT73E19
GmUGT9

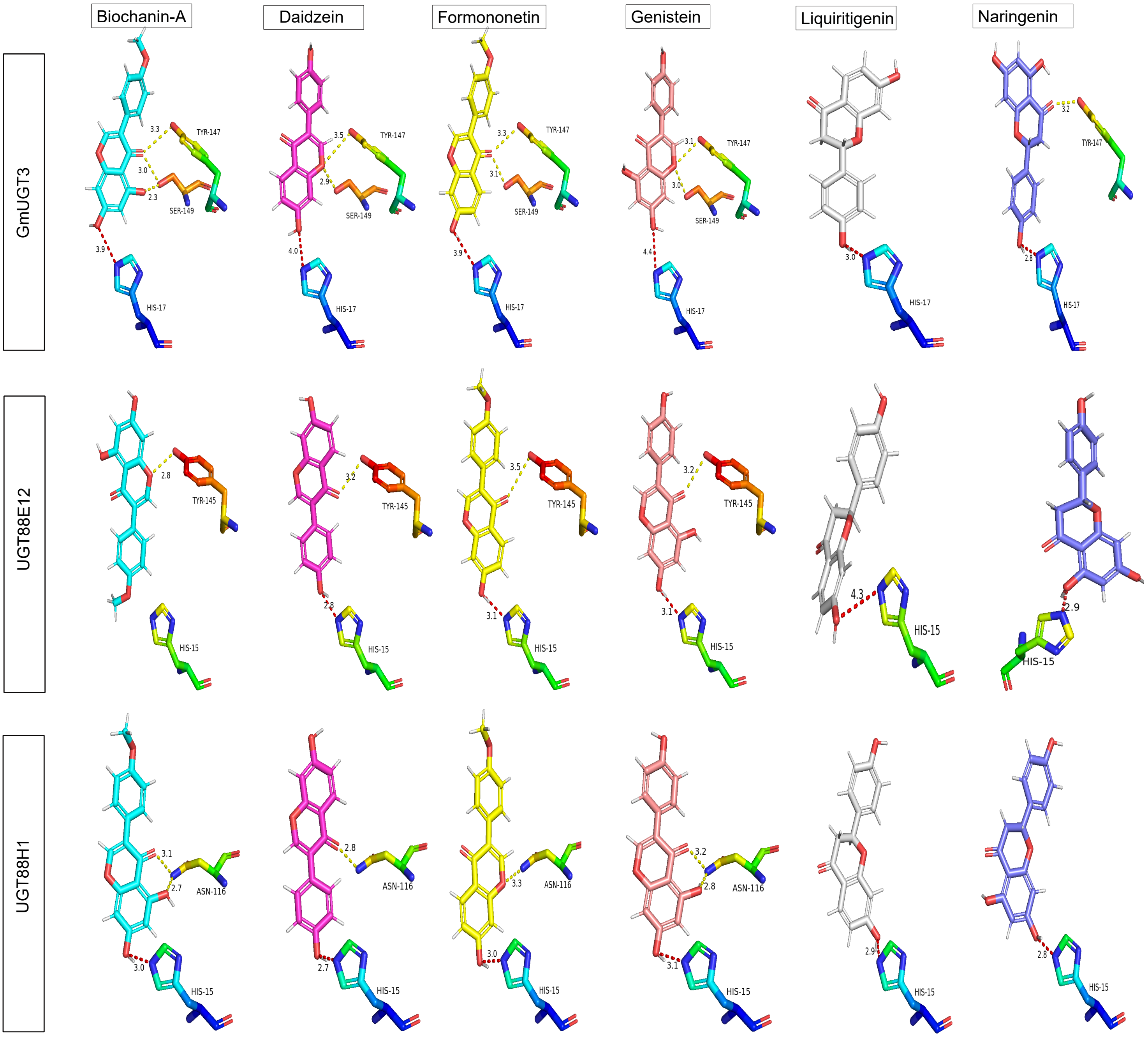

Figure S5A: Docking results of Group 1 UGTs with selected substrates. Different orientations of catalytic His highlights distinct active site structures that results in different orientation of substrates (the substrates are shown in vertical orientations for better visuals and comparison). Yellow dotted lines represent polar interactions between substrates and amino acids, red dotted lines represent the distance between catalytic His and the closest OH group. The distance is given in Å units. Key amino acids and substrates are shown in stick models. The carbon skeleton of biochanin-A, daidzein, formononetin; genistein, liquiritigenin and naringenin is coloured cyan, pink, yellow, salmon, grey, and slate respectively, and the oxygen atoms are coloured red.

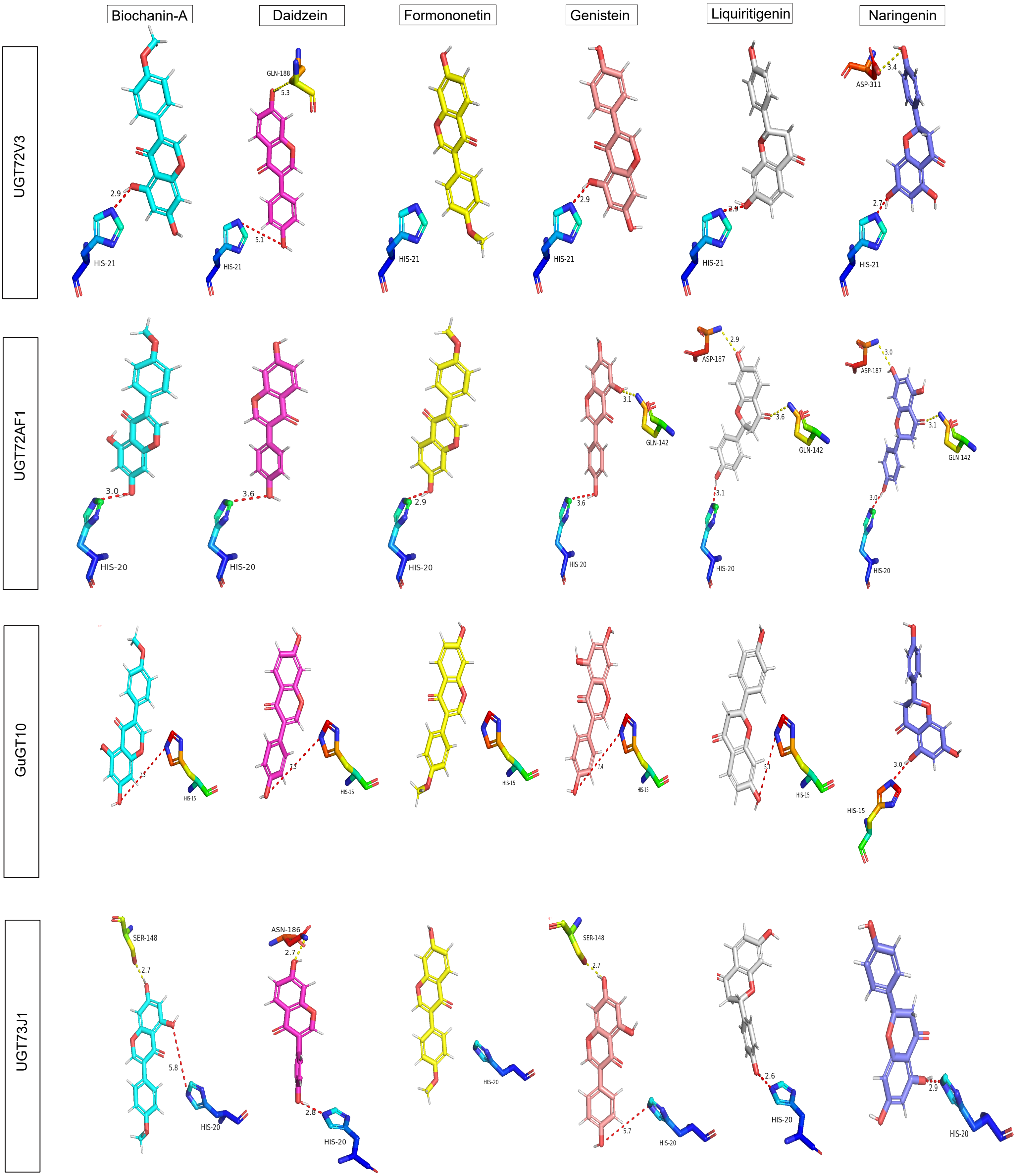
Biochanin-A
Daidzein
Formononetin
Genistein
Liquiritigenin
Naringenin
UGT72V3
UGT72AF1
GuGT10
UGT73J1
HIS-21
GLN-188
ASP-311
HIS-20
ASP-187
GLN-142
HIS-15
SER-148
ASN-186

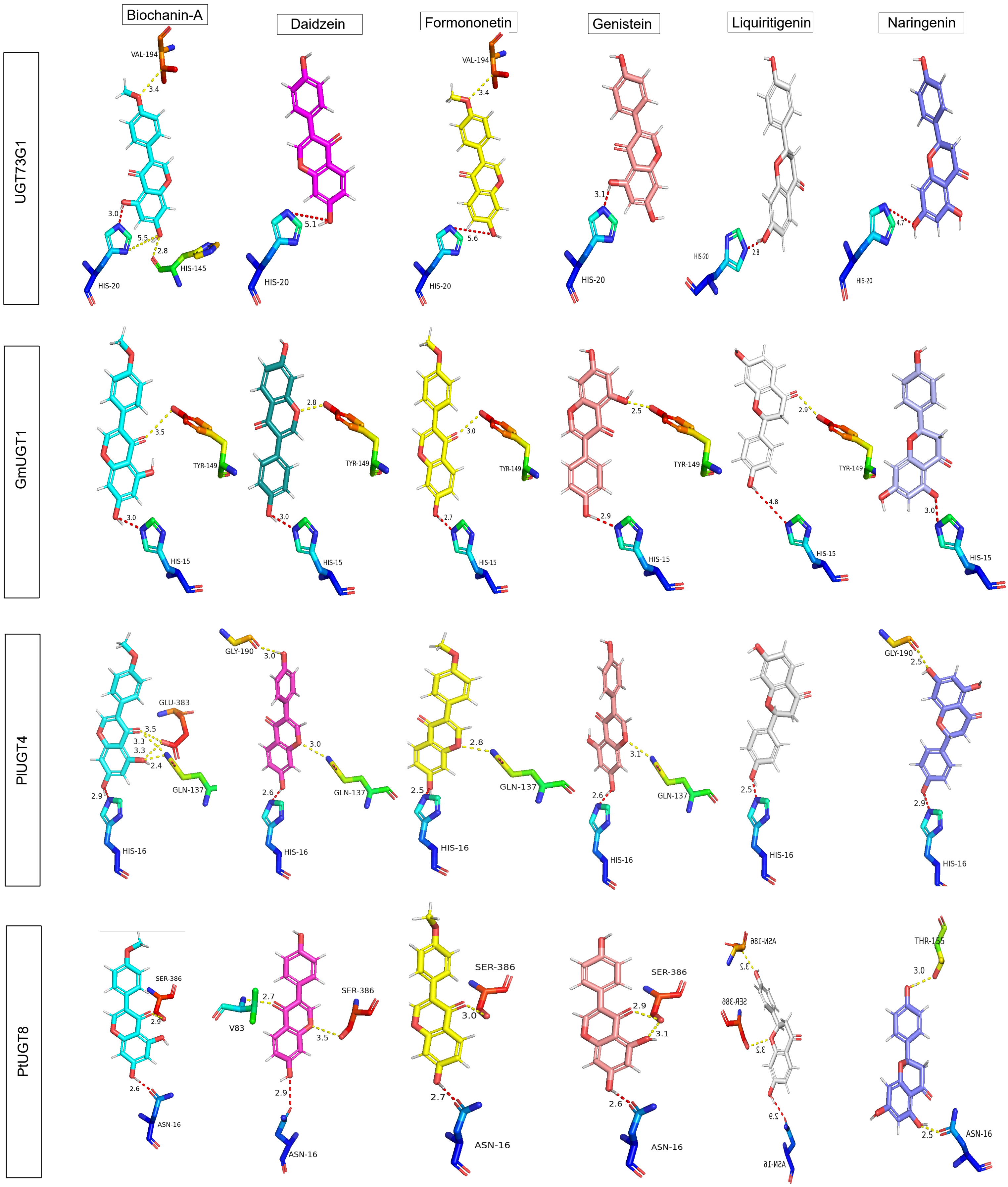

Figure S5B: Docking results of Group 2 UGTs with selected substrates. Different orientations of catalytic His highlights distinct active site structures that results in different orientation of substrates (the substrates are shown in vertical orientations for better visuals and comparison). Yellow dotted lines represent polar interactions between substrates and amino acids, red dotted lines represent the distance between catalytic His and the closest OH group. The distance is given in Å units. Key amino acids and substrates are shown in stick models. The carbon skeleton of biochanin-A, daidzein, formononetin; genistein, liquiritigenin and naringenin is colored cyan, pink, yellow, salmon, grey, and slate respectively, and the oxygen atoms are colored red.

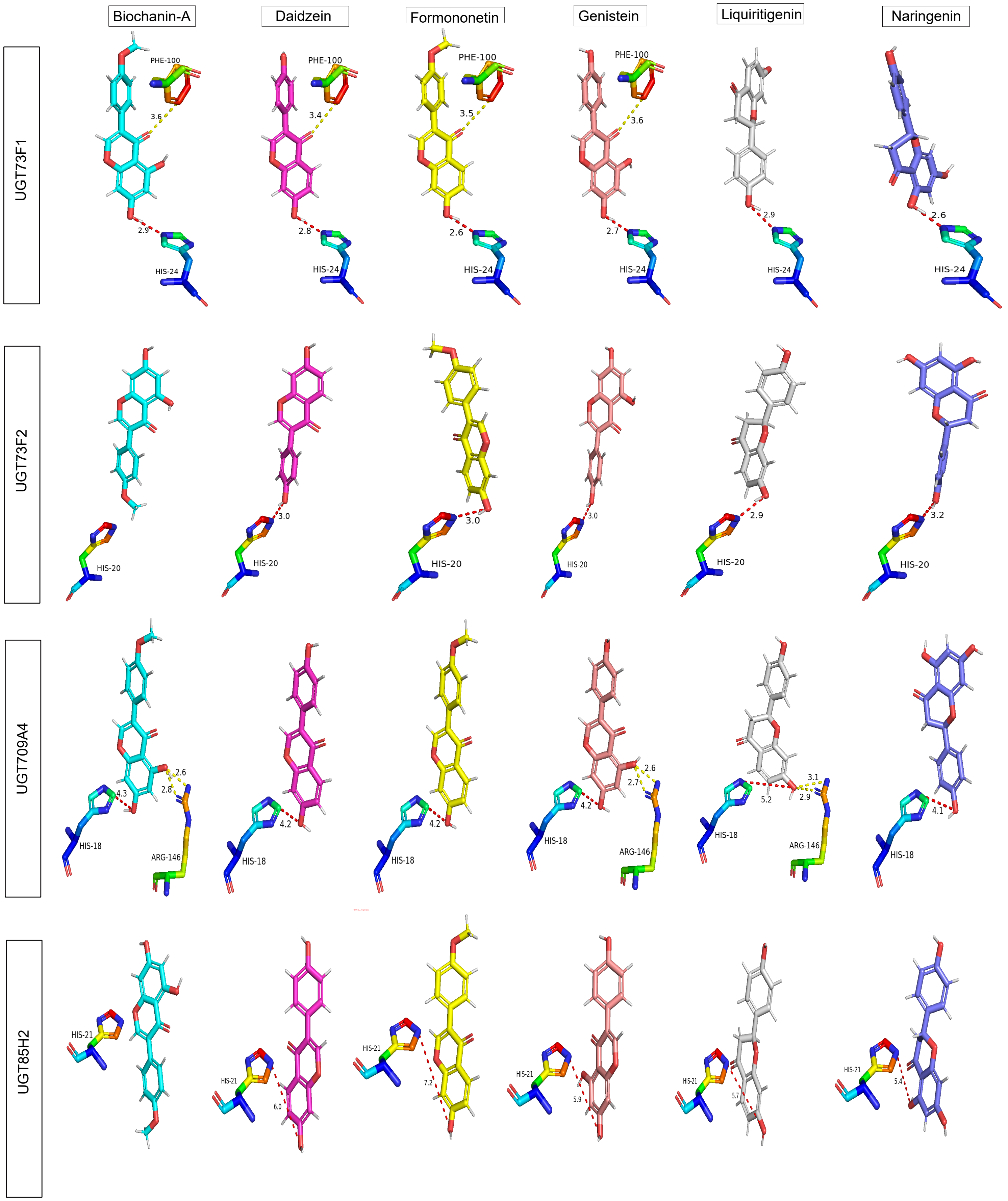

Biochanin-A
Daidzein
Formononetin
Genistein
Liquiritigenin
Naringenin
UGT73F1
PHE-100
HIS-24
UGT73F2
HIS-20
UGT709A4
HIS-18
ARG-146
UGT85H2
HIS-21

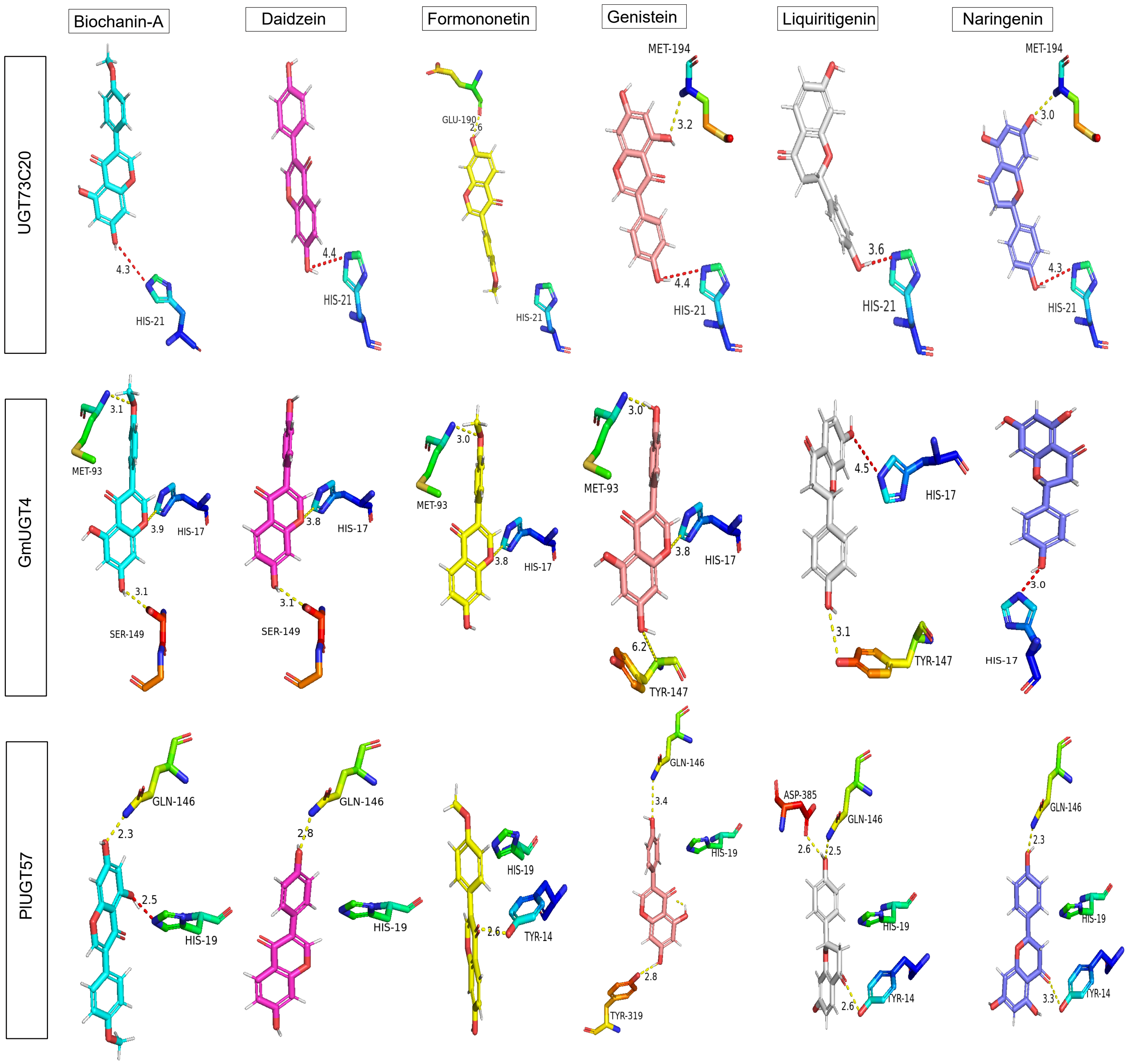

Figure S5C: Docking results of Group 3 UGTs with selected substrates. Different orientations of catalytic His highlights distinct active site structures that results in different orientation of substrates (the substrates are shown in vertical orientations for better visuals and comparison). Yellow dotted lines represent polar interactions between substrates and amino acids, red dotted lines represent the distance between catalytic His and the closest OH group. The distance is given in Å units. Key amino acids and substrates are shown in stick models. The carbon skeleton of biochanin-A, daidzein, formononetin; genistein, liquiritigenin and naringenin is coloured cyan, pink, yellow, salmon, grey, and slate respectively, and the oxygen atoms are coloured red.

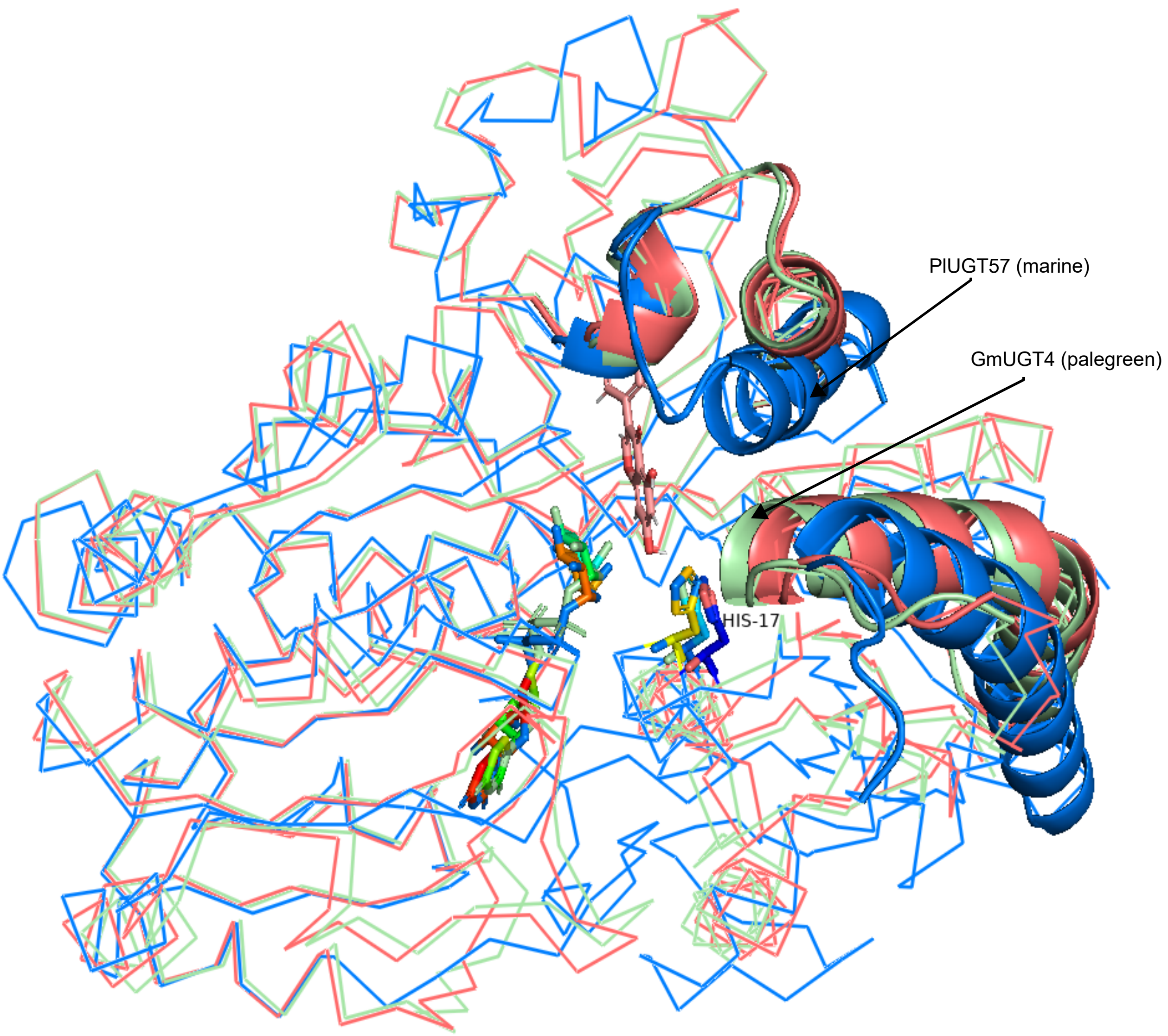

Figure S6: Structural comparison of PlUGT15 (model UGT, deep salmon colour), PlUGT57 (marine colour) and GmUGT4 (pale green colour). The extended loop regions (shown by the arrowhead) do not let substrates move deep in the acceptor binding cavity (active site), thus the substrate either stays on the surface or accesses the active site through different channels. Protein models of PlUGT57 and GmUGT4 were aligned with PlUGT15 (model UGT) due to the similarity between the models (based on RMSD values) in PyMOL.
